# Supplementary material for: Association of N, N-diethyl-m-toluamide (DEET) with arthritis among adult participants
Source: PLoS One. 2024 Dec 31;19(12):e0313012. doi: 10.1371/journal.pone.0313012 (PMC11687794; doi:10.1371/journal.pone.0313012)
Supplement: Attachment — Submitted filename: Response to Reviewers.docx [file pone.0313012.s001.docx]

Reply to Reviewer 1:

Q1. NHANES is a cross-sectional survey, and the incidence rate cannot be calculated. In this paper, incidence rate is used to replace the prevalence rate for many times, please check.

**Reply:**

Thank you for underlining this deficiency. We apologize for any inconvenience caused by our inappropriate expression.

Changes in the text:

This phrase was modified according to the comment (Line 33, 43, page 2; Line 70, 73, page 3; Line 151, page 6; Line 251, 267, page 11).

Q 2. The result expression is inconsistent with the table content. See Table 2. "Univariate analyses suggest that family income and BMI exhibit no association with sleep disorder prevalence"

**Reply:**

Thank you for underlining this deficiency. We apologize for any inconvenience caused by our inappropriate expression. This is an expression error. The correct term is 'arthritis prevalence', not 'sleep disorder prevalence'. Furthermore, based on Table 2, we have revised the description of the results section in the main text.

Changes in the text:

This phrase was modified according to the comment (Lines 166-168, page 7).

Q 3. What is the result of "sleep disorder" on single-factor analysis, as shown in Table 2 on the prevalence of "no association" and on the prevalence of risk factors?

**Reply:**

Thank you for underlining this deficiency. We apologize for any inconvenience caused during your review. The results of univariate analysis show that sleep disorders are associated with the risk of arthritis.

Changes in the text:

This phrase was modified according to the comment (Line 170, page 7).

Q 4. The wording of covariates in the table should be consistent in the text

**Reply:**

Thank you for underlining this deficiency. We have unified the descriptions of covariates across the manuscript and tables.

Changes in the text:

This phrase was modified according to the comment (Lines 129-135, pages 5-6).

Q 5. Figure 2 and Figure 3 are marked backwards

**Reply:**

Thank you for underlining this deficiency. We have re-tagged the figures in order.

Q 6. In Figure 2, "non-Mexican Hispanic males between the ages of 45-59", P=0.007, indicating a statistical difference, but OR 95%CI(0.917,5.599) showed no statistical difference, please check

**Reply:**

Thank you sincerely for your suggestion, which is very important for improving our manuscript. We conducted a reanalysis of the data and found that in Figure 2, there was no statistically significant difference (P=0.076) among non Hispanic males aged 45-59.

Changes in the figure 2:

Q 7. Why is there no group of 20-44 years old in this group, please explain or add

**Reply:**

Thank you for underlining this deficiency. We conducted statistical analysis using the group of 20-44 years old as a reference and redrawn Figure 2.

Changes in the figure 2:

Q 8. Why not do other types of grouping? Please explain or add

**Reply:**

Thank you for underlining this deficiency. The reason for selecting race, age, and gender as stratified variables is mainly because these three demographic variables have statistical significance in other DEET related studies, and our study is no exception. In fact, there are many covariates in our study that have shown statistical significance, but since there are no other literature reports, we cannot make more choices. If all statistically significant variables in the univariate analysis are included, the data in the stratified analysis will increase exponentially and have no reference value.

(Cui Q, Zhu X, Guan G, Hui R, Zhu L, Wang J: Association of N,N-diethyl-m-toluamide (DEET) with obesity among adult participants: Results from NHANES 2007-2016. Chemosphere 2022, 307(Pt 1):135669; Haleem ZM, Yadav S, Cushion ML, Tanner RJ, Carek PJ, Mainous AG: Exposure to N,N-Diethyl-Meta-Toluamide Insect Repellent and Human Health Markers: Population Based Estimates from the National Health and Nutrition Examination Survey. Am J Trop Med Hyg 2020, 103(2):812-814; Wu R, Zhu X, Xing Y, Guan G, Zhang Y, Hui R, Cui Q, Liu Z, Zhu L: Association of N, N-diethyl-m-toluamide (DEET) with hyperuricemia among adult participants. Chemosphere 2023, 338:139320.)

Q 9. In logistics, Q1 is the reference group, and the reference groups from Q2 to Q4 are all Q1. Q1 does not have P, or P is P for trend

**Reply:**

Thank you for underlining this deficiency. We have modified our text as advised. We will use a '-' in the 'p' row of Q1 in Tables 3-5 to indicate 'no p'

Changes in the tables 3-5.

Q 10. The description in table34 does not match the table. Please check and correct it

**Reply:**

Thank you for underlining this deficiency. We have modified our text as advised.

Changes in the text:

These sentences were modified according to the comment (Lines 172-179, 186-195, pages 7-8).

Q 11. The table is unified, and the data in Q1 is either blank or REF is added

**Reply:**

Thank you for underlining this deficiency. We have modified our text as advised. We added "ref" in the blank field of the Q1 data.

Changes in the tables 3-5.

Q 12. The description of quartile is wrong, there is no "quantiles (Q1-Q2)" type, there is only "Q1" quantiles=Q, and the data has been changed into Q1, Q2, Q3 and Q4 in the logistics analysis, which is either a continuous variable or a grouping variable, and there is no expression of Q1-Q2. Please carefully study the significance of data analysis methods and the description of results, and then revise the description of results analysis in the paper.

Q 13.RCS diagram is not clear, please adjust it

**Reply:**

Thank you for underlining this deficiency. We have generated a clearer RCS diagram.

Changes in the figure 3.

Q 14. N, n-diethyl-meta-toluamide is covered at length in the first three paragraphs of the discussion, but is not relevant to this article. The first paragraph is suggested to reduce the length and take the essence of it. It is suggested to shorten these three paragraphs into one paragraph.

**Reply:**

Thank you for underlining this deficiency. We have modified our text as advised. We have streamlined the information on DEET and incorporated it into the introductory section.

Changes in the text:

These sentences were modified according to the comment (Lines 52-54, page 2; Lines 55-60, pages 2-3).

Q 15. It is suggested that paragraphs 4, 5 and 6 summarize the results of the whole paper as the beginning of the discussion, emphasizing the innovation and value of this study, but reducing the length and condensing the sentences.

**Reply:**

Thank you for underlining this deficiency. We have modified our text as advised.

Changes in the text:

These sentences were modified according to the comment (Lines 223-230, pages 9-10).

Q 16. Increase the comparison between this study and others' studies, as well as possible mechanism explanations for the results found in this study

**Reply:**

Thank you for underlining this deficiency. We have modified our text as advised. We have added a discussion on relevant research and explored two possible mechanisms by which DEET affects the risk of arthritis (affecting inflammatory response and mitochondrial function). However, due to the limited research on DEET, further comparison and discussion cannot be conducted.

Changes in the text:

These sentences were modified according to the comment (Lines 231-239, page 10).
